# Knowledge, attitude and practice towards COVID-19 among health professionals in Ethiopia: A systematic review and meta-analysis

**DOI:** 10.1371/journal.pone.0247204

**Published:** 2021-02-19

**Authors:** Eyasu Alem Lake, Birhanu Wondimeneh Demissie, Natneal Atnafu Gebeyehu, Addisu Yeshambel Wassie, Kelemu Abebe Gelaw, Gedion Asnake Azeze

**Affiliations:** 1 Department of Nursing, College of Health Science and Medicine, Wolaita Sodo University, Wolaita Sodo, Ethiopia; 2 Department of Midwifery, College of Health Science and Medicine, Wolaita Sodo University, Wolaita Sodo, Ethiopia; University Magna Graecia of Catanzaro, ITALY

## Abstract

**Background:**

The World Health Organization (WHO) declared coronavirus disease 2019 (COVID-19) a global pandemic on 11th March, 2020. In Ethiopia, more than 90,490 and 1,300 confirmed cases and deaths were reported by the Federal Ministry of Health at the time of writing up this project. As health care providers are frontline workers managing the COVID-19 pandemic, this systematic review and meta-analysis aimed to assess the pooled level of knowledge, attitude, and practice towards COVID-19 among health professionals in Ethiopia.

**Methods:**

PubMed, Google Scholar, Excerpta Medica database (EMBASE), Cochrane Library, Web of Science, and African Journal of Online (AJOL) were searched. The data were extracted using Microsoft Excel and analyzed using STATA version 14. Publication bias was checked by funnel plot and more objectively through Egger’s regression test, with P < 0.05 considered to indicate potential publication bias. The heterogeneity of studies was checked using I^2^ statistics. Pooled analysis was conducted using a weighted inverse variance random-effects model. Subgroup analysis was done related to geographic region and time. A leave-one-out sensitivity analysis was also employed.

**Result:**

A total of 11 studies with 3,843 study participants for knowledge, eight studies with 2,842 study participants for attitude and 10 studies with 3, 435 study participants for practice were used to estimate the pooled level of good knowledge, positive attitude and poor practice among health professionals. The overall estimated good level of knowledge, positive attitude and poor practice towards COVID-19 was found to be 79.4% (95% CI: 73.5%-85.2%; I2 = 96%), 73.7% (95%CI: 63.09%-84.4%; I2 = 98.3%) and 40.3% (95%CI: 31.1%-49.6%; I2 = 97.1%) respectively.

**Conclusion:**

Study findings showed that there were significant gaps in COVID-19 related knowledge, attitude and practice with respect to World Health Organization recommendations on COVID-19 management and personal protection practices. This study therefore recommends that institutions provide with immediate effect accurate and up-to-date information on COVID-19 and training that encourages improved knowledge, attitude and practice to mitigate this pandemic.

## Introduction

A cluster of atypical pneumonia cases was reported to the World Health Organization (WHO) in Wuhan, China, on 31^st^ December 2019 [1]. The name given to this disease was Corona Virus Disease-19 (COVID-19). On March 11, 2020, WHO declared that the disease was a global pandemic [2]. At the time of writing (21^th^ October 2020), COVID-19 had spread to 217 countries and territories and accounted for 41,053,557 confirmed cases of COVID-19 and 1,129,775 deaths. In Ethiopia, there were 90,490 confirmed cases and 1,371 deaths [3]. COVID-19 is a new emerging respiratory disease caused by a single-strand, positive-sense ribonucleic acid (RNA) virus called Severe Acute Respiratory Syndrome Corona Virus-2 (SARS-CoV-2) [4].

There is no known cure for this infection and at the time of writing there were no vaccines available for routine use. The main intervention is the use of preventive measures as recommended by experts which include: use of face masks, wearing gloves, frequent hand washing, avoiding frequent exposure to sensitive areas, keeping physical distance, and avoidance of touching the nose, mouth, and eye with contaminated hands or other contaminated materials [5]. Health care professionals, because of close care needed to look after infected patients, are at serious risk of infection [6].

The World Health Organization (WHO) has issued a number of guidelines on COVID-19 to various sectors of society, and has provided a range of education and training materials to health care workers (HCWs) to increase their awareness and preparedness for COVID-19 control and prevention [7]. While HCWs are at high risk of infection because of working with infected patients in overcrowded hospitals, that in resource-limited settings include a lack of isolation rooms, it is possible that they also have inadequate awareness of infection prevention practices [8]. Incorrect attitudes and poor practices may directly increase the risk of infection among HCWs [9].

Although HCWs represent less than 3% of the population in most countries, especially in low- and middle-income countries, 14% of COVID-19 victims are HCWs, according to the World Health Organization report [10]. The number could rise as much as 35% in some countries. There are still questions about access to information and the quality of information, and it is often difficult to ascertain whether HCWs are infected in the workplace or in the area where they live [10]. The published literature suggests that lack of knowledge and misunderstandings among HCWs leads to delayed diagnosis, spread of disease and poor infection control practice. Several thousand HCWs have already been infected, mainly in China [6]. Preventing nosocomial transmission of this communicable disease is therefore a priority.

To our knowledge, there are only a few studies on Knowledge, Attitude and Practice (KAP) with respect to COVID-19 among HCWs in Ethiopia, and these have shown significant differences in KAP between the various regions of the country. A review done by Samuel SC et al showed that disease transmission increased because the population was reluctant to implement directives from the government [11]. However, this study did not provide a compressive estimation of KAP to COVID-19 among health professionals. We therefore conducted this systematic review and meta-analysis to provide an estimate of KAP with respect to COVID-19 among Ethiopian HCWs for the purpose of improving program planning and interventions focused on the prevention and control of this global pandemic.

## Methods

### Searching strategy and source of information

This study was conducted to estimate the pooled level of KAP towards COVID 19 among HCWs in Ethiopia. We checked the DARE database (http://www.library.UCSF.edu) and the Cochrane library to ensure this had not been done before and to avoid duplication. We also checked whether there was any similar ongoing systematic review and meta-analysis in the PROSPERO database ((PROSPERO 2017:CRD42017074407); Available from http://www.Crd.york.ac.uk/ PROSPERO_REBRANDING/ display record. asp? ID = CRD42017074407. These checks reassured us that there had been no previous similar studies undertaken.

All relevant and published researches in the following databases; PubMed, Google Scholar, Excerpta Medica database (EMBASE), Cochrane Library, Web of Science, and African Journal of Online (AJOL) were searched. We reviewed grey literature using Google. Unpublished studies were sought from the official website of an international and/or local organization or university.

The following core search terms or phrases were used; knowledge, awareness, attitude, perception, practice, pandemic, COVID-19, SARS CoV-2, coronavirus, health professional and health care provider. Search terms were pre-defined to allow a complete search strategy that included all-important studies. All fields within records and MeSH (Medical Subject Headings) and Boolean operators were used to search in the advanced PubMed search engine.

Notably, to fit with the advanced PubMed database the following search strategy was developed using different Boolean operators; ((((((Knowledge[tw] OR Awareness[tw] OR attitude[tw] OR perception[tw] OR practice[tw])) OR ("Health Knowledge, Attitudes, Practice"[Mesh] OR "Practice Management, Veterinary"[Mesh] OR "Knowledge Discovery"[Mesh] OR "Knowledge Management"[Mesh] OR "Practice Patterns, Nurses‴[Mesh] OR "Knowledge"[Mesh]))) AND (((Pandemic[tw] OR COVID-19[tw] SARS CoV-2[tw] OR corona virus[tw])) OR ("COVID-19 vaccine" [Supplementary Concept] OR "pediatric multisystem inflammatory disease, COVID-19 related" [Supplementary Concept] OR "COVID-19 serotherapy" [Supplementary Concept] OR "COVID-19 diagnostic testing" [Supplementary Concept] OR "ORF7b protein, SARS-CoV-2" [Supplementary Concept] OR "ORF3a protein, SARS-CoV-2" [Supplementary Concept] OR "ORF1ab polyprotein, SARS-CoV-2" [Supplementary Concept] OR "nucleocapsid protein, Coronavirus" [Supplementary Concept] OR "spike protein, SARS-CoV-2" [Supplementary Concept]))) AND (((Health care workers[tw] OR health professional [tw])) OR ("Education, Public Health Professional"[Mesh] OR "Allied Health Personnel"[Mesh]))) AND Ethiopia[tw]. We reviewed studies that assessed KAP on COVID-19 through face to face interviews, self-administered questionnaires or electronically administered structured questionnaires among HCWs.

### Measurements of KAP

#### Knowledge

Knowledge was assessed based on 16 questions about COVID-19 that included the causative agent, clinical signs, symptoms, mode of transmission, treatment and vaccine availability and mechanisms of prevention. Knowledge was defined as good if the respondents scored above the mean level.

#### Attitude

Attitude was assessed by using 11 questions on COVID-19 control, attitude towards preventive measures, fear of acquiring the disease and interest in participating in COVID-19 patient care. A respondent who scored above the mean level was defined as having a positive attitude.

#### Practice

Practice was assessed by using 15 questions about protective measures for COVID-19 and the respondent was categorized as showing poor practice if he/she scored below the mean.

### Reporting

The results of this review were reported in line with the Preferred Reporting Items for Systematic Review and Meta-Analysis statement (PRISMA) guideline [12].

### Eligibility criteria

All observational studies on KAP towards COVID-19 were considered for this study. Those studies about KAP among HCWs which were published in English were included and there was no restriction on study period or type of HCW. The level of good knowledge, positive attitude and poor practice was calculated using the data presented in the studies. Papers were excluded if they were: review articles, studies reporting confused data or with probable errors, studies without any information on the country and studies which were not able to fully access. An attempt was made to contact the corresponding authors using the email address or phone number as provided in the published articles.

### Study selection and extraction

Retrieved articles were exported to the reference manager software, Mendeley Desktop, and this was used to remove duplicate studies. Three independent reviewers screened the title and abstract. Any disagreement was handled based on established article selection criteria. Data were extracted using a standardized data extraction format prepared in Microsoft Excel by two independent reviewers. Any discrepancy during extraction was solved through discussion. The name of the first author, study area and region, the study month and year, the study design, year of publication, study population, sample size, response rate and level of good knowledge, positive attitude and poor practice were collected.

### Quality assessment

Three independent authors appraised the quality of the studies. The Joanna Briggs Institute (JBI) quality appraisal checklist was used [13]. When there was disagreement, all three authors discussed and resolved the issue. The critical appraisal checklist had 8 parameters with options of “yes, no, unclear and not applicable.” The quality parameters included the following questions: (1) Were the criteria for inclusion in the sample clearly defined?, (2) Were the study subjects and the setting described in detail?, (3) Was the exposure measured in a valid and reliable way?, (4) Were objective, standard criteria used for measurement of the condition?, (5) Were confounding factors identified?, (6) Were strategies to deal with confounding factors stated?, (7) Were the outcomes measured in a valid and reliable way?, and (8) Was an appropriate statistical analysis used?. Studies were considered low risk if there was a score of 50% and above of the quality assessment indicators.

### Statistical analysis

The data were extracted using Microsoft Excel and analyzed by using STATA version 14 statistical software (stataCorp LP, 4905 Lakeway Drive, College Station, TX 77845, USA). Publication bias was checked by funnel plot and more objectively through Begg and Egger’s regression tests, with P < 0.05 considered to indicate potential publication bias [14, 15]. The presence of significant between-study heterogeneity was assessed using the Cochrane Q statistic. I^2^ was used to quantify between-study heterogeneity, in which a value of 0%, 25%, 50%, and 75% indicated no, low, medium, and increased heterogeneity, respectively [16]. A forest plot was used to visualize the presence of heterogeneity. Since we found a high level of heterogeneity, we used a random-effect model for analysis to estimate Der Simonian and Laird’s pooled effect. Subgroup analysis was done by stud region and month. A leave-one-out sensitivity analysis was employed to see the effect of a single study on the overall meta-analysis estimate. The results were presented in the form of text, tables and figures.

## Results

### Search outcomes

**Fig 1:**
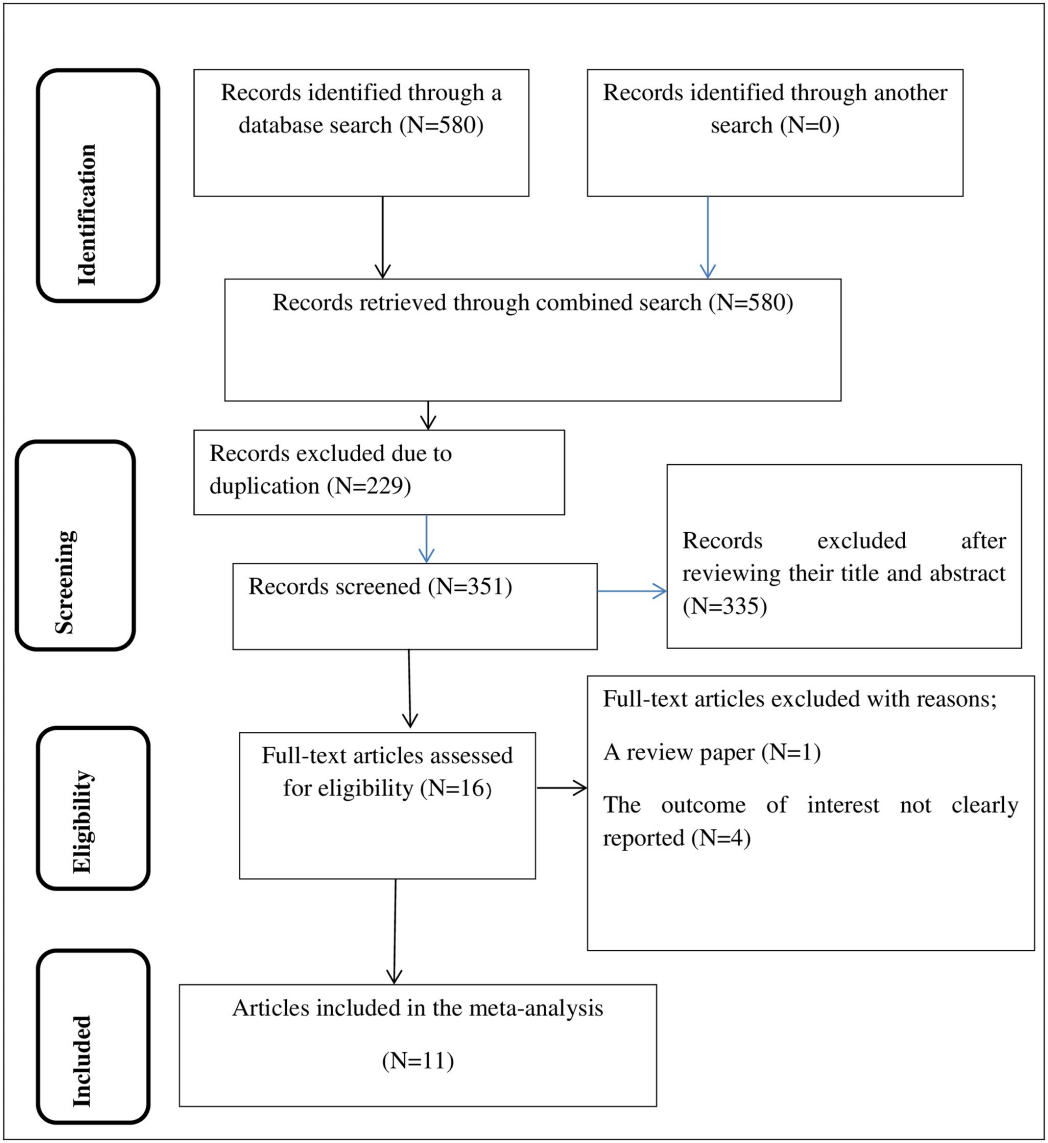
Schematic presentation of study selection for systematic review and meta-analysis of KAP towards COVID-19 among health professionals in Ethiopia.

There were 580 articles retrieved using the electronic search. Of these articles, 229 were excluded due to duplication and 16 articles were fully accessed and assessed for qualification. Eventually, 11 articles met the eligibility criteria and were included in the final meta-analysis. Among those 11 eligible articles, seven of them assessed all KAP characteristics towards COVID-19 (Fig 1).

### Characteristics of included studies

Eleven articles which assessed knowledge, 10 which assessed practice and 8 which assessed attitude, all of which fulfilled the inclusion criteria, were included in this systematic review and meta-analysis. All studies had a low risk during the quality assessment. Three studies were conducted in the Amhara region [17–19], two in Addis Ababa [20, 21], one in Oromia [22], one in Tigray [23], one in SNNP [24] and one in Harari [25]. The other two articles on KAP and COVID-19 were nationwide studies [26, 27].

All studies were conducted in 2020 from February to June. All studies employed a cross-sectional study design using a face-to-face or electronically administered questionnaire. The sample size ranged from 166 to 532, and the response rate ranged from 84.3% to 100%. The highest level of good knowledge (93.3%) was recorded in a study from Oromia region and the lowest (53.2%) was recorded in a study conducted in Addis Ababa. There was a high level of poor practice recorded in a study done in Addis Ababa which was 70.2% (Table 1).

**Table 1:** Characteristics of studies included in the systematic review and meta-analysis on level of knowledge, attitude and practice towards COVID-19 among health professionals, Ethiopia.

*Authors name*	Sampling month /year	Date of accessed	Study Area	Study Region	Study design	Sample size	Good level of knowledge % (95%CI)	Positive Attitude %(95% CI)	Poor practice%(95%CI)	Study quality
Tesfaye et al	March/2020	20/10/2020	Addis Ababa	Addis Ababa	Cross-sectional	295	53.2	89.8	70.2	Low risk
Asemahagn	April /2020	20/10/2020	Amhara Region	Amhara	Cross-sectional	398	70		38%	Low risk
Kassie BA	March /2020	20/10/2020	Central Gondar	Amhara	Cross-sectional	408	73.8	65.7		Low risk
Abebe Habtamu Tamire	April /2020	15/10/2020	Addis Ababa	Addis Ababa	Cross-sectional	526	87.1	74.9	32.37	Low risk
Arif Husswn	February/2020	15/10/2020	Jugal	Harar	Cross-sectional	207	79.74	75.53	24.7	Low risk
Bedru Jemal		11/10/2020	Nationwide	Nationwide	Cross-sectional	397	88.2	94.7	36.5	Low risk
Tadesse DB	March /2020	12/10/2020	Aksum	Tigray	Cross-sectional	415	74	72	33	Low risk
Girma et al	May /2020	12/10/2020	Selected hospitals	Nationwide	Cross-sectional	273	83.11		31.82	Low risk
Abera Mersha	June/2020	08/10/2020	Gamo zone	SNNP	Cross-sectional	428	84.10	53	64.7	Low risk
Dereje Tsegaye	April /2020	08/10/2020	Ilu Abba Bor and Buno Bedelle	Oromia	Cross-sectional	330	93.3		35.8	Low risk
Dejen Getaneh	June2020	08/10/2020	South Gondar	Amhara	Cross-sectional	166	84.9	63.3		Low risk

### Publication bias

**Fig 2:**
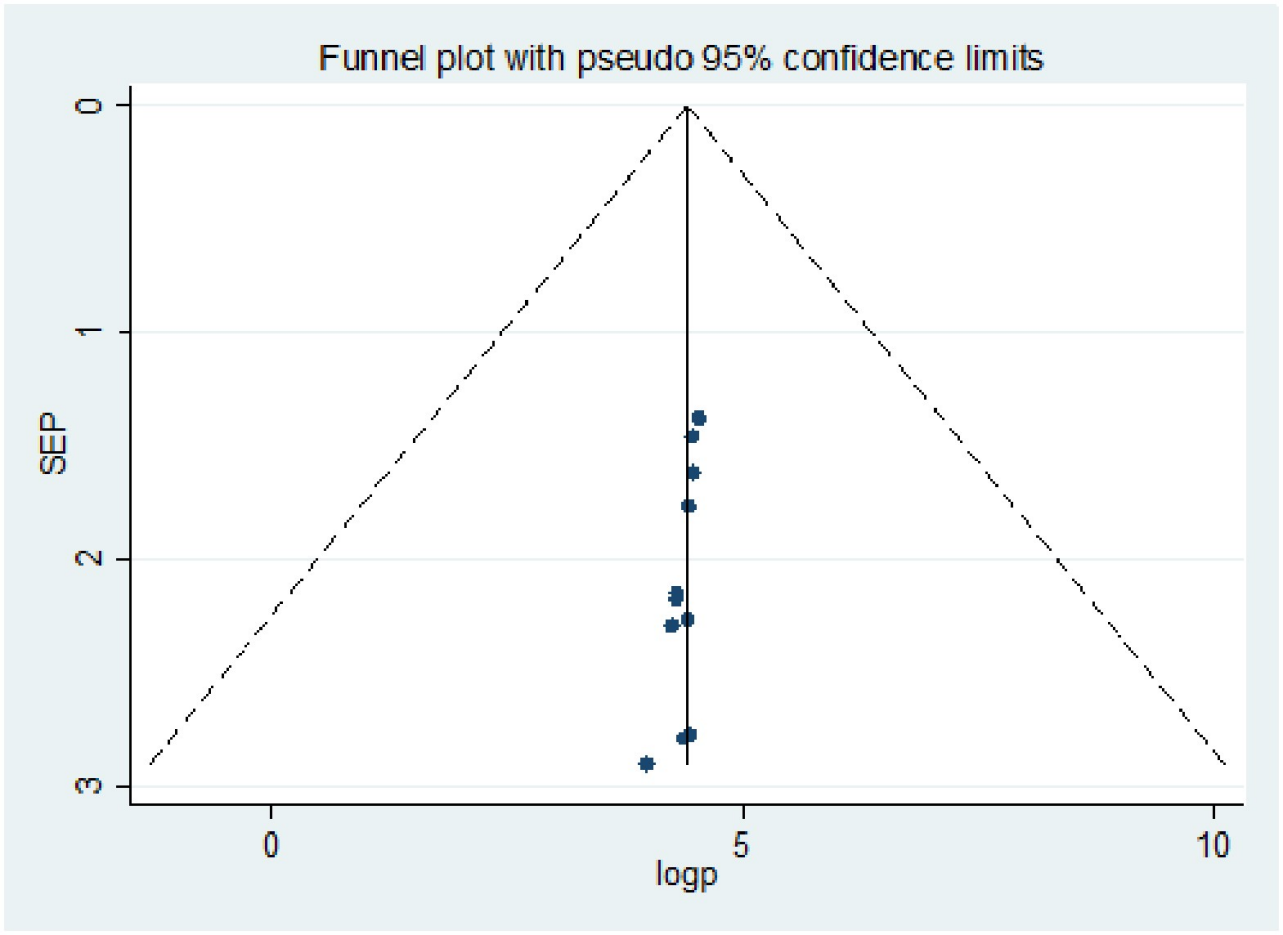
Funnel plots for publication bias of level of knowledge towards COVID-19 among health professionals in Ethiopia.

**Fig 3:**
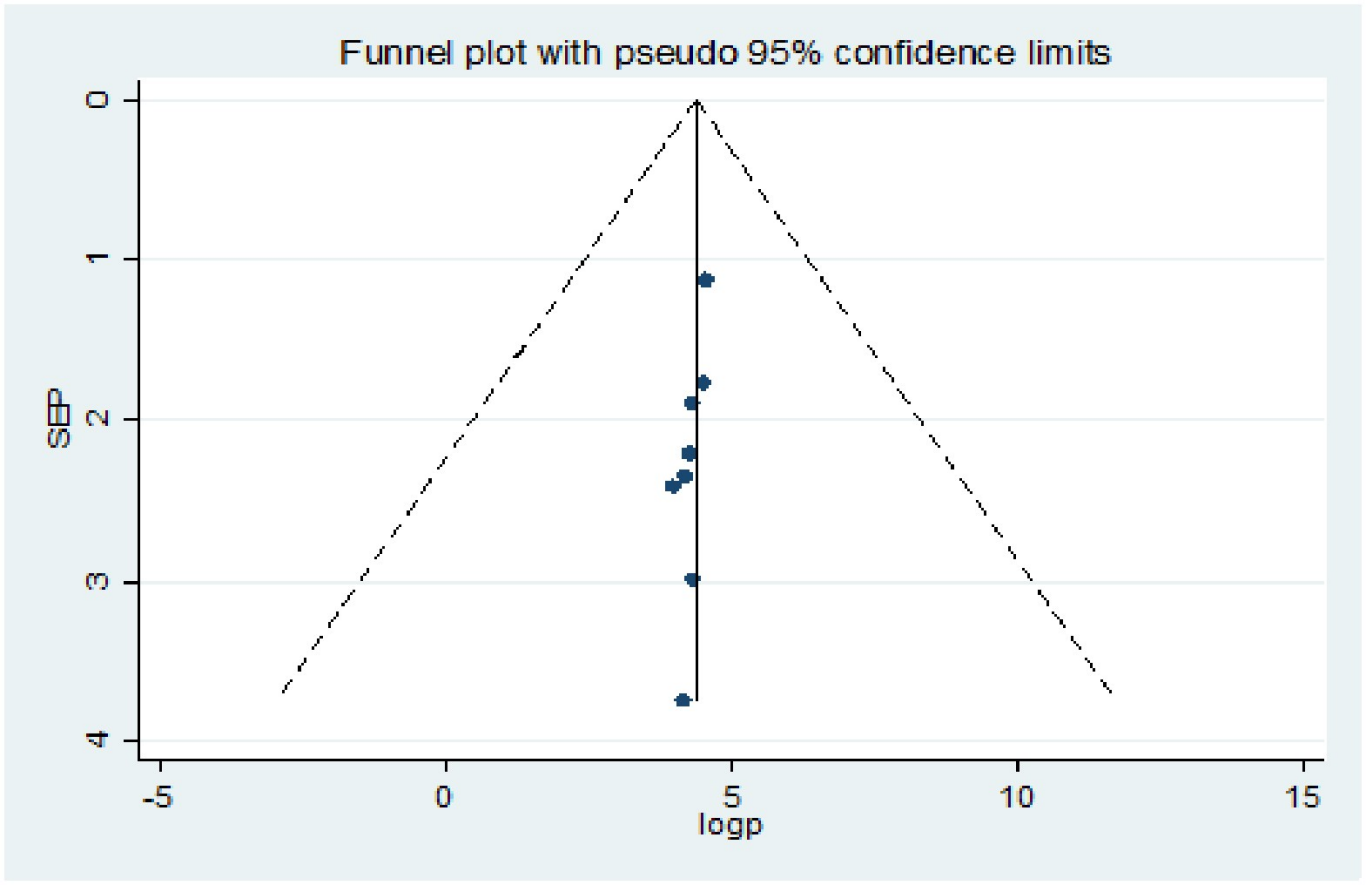
Funnel plots for publication bias of level of positive attitude towards COVID-19 among health professionals in Ethiopia.

**Fig 4:**
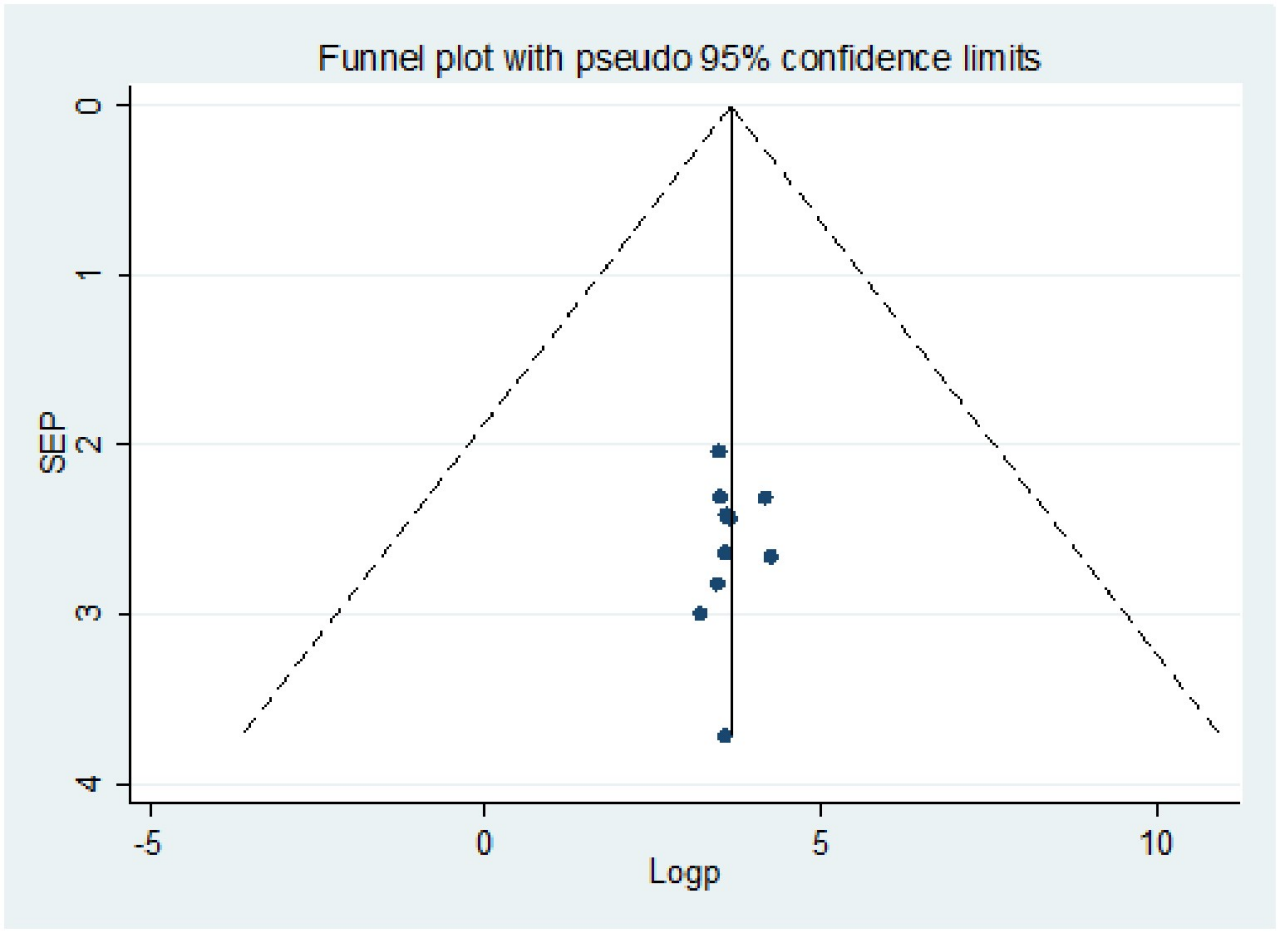
Funnel plots for publication bias of level of poor practice towards COVID-19 among health professionals in Ethiopia.

Publication bias was assessed using a funnel plot and the Egger and Begg regression test at P<0.05. There was statistical evidence of publication bias for a good level of knowledge. A funnel plot showed some asymmetrical distribution, the Begg and Egger tests were statistically significant with P-values = 0.013 and = 0.027 respectively (Fig 2). There was statistical evidence of publication bias for positive attitude. A funnel plot showed some asymmetrical distribution, the Begg and Egger tests were statistically significant with P-values = 0.0063 and = 0.019 respectively (Fig 3). There was statistical evidence of publication bias for poor practice using a funnel plot and the Egger, and Begg regression test (Fig 4).

### Level of knowledge, attitude and practice towards COVID-19

**Fig 5:**
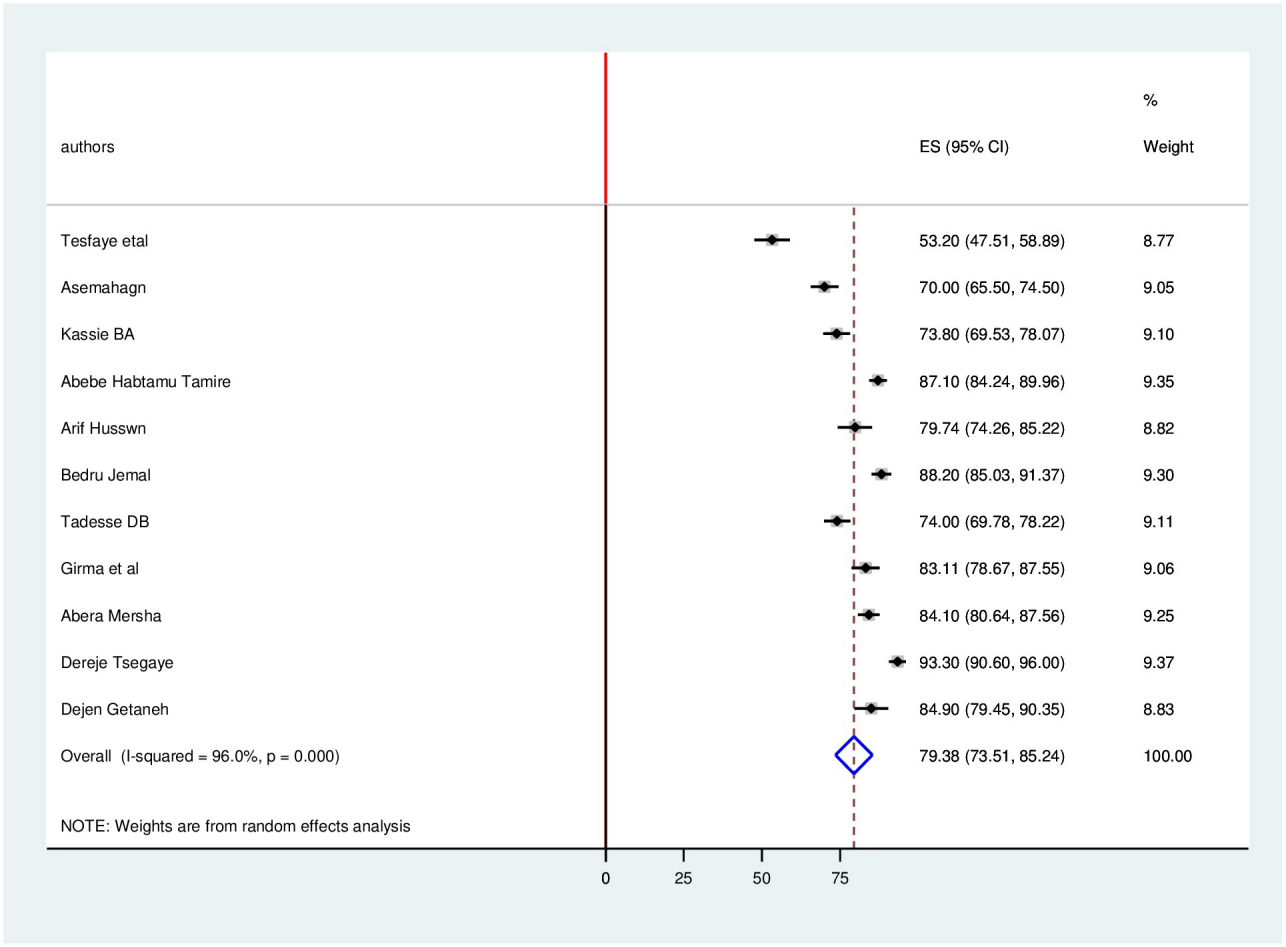
Forest plot for the pooled level of good knowledge towards COVID-19 among health professionals in Ethiopia.

**Fig 6:**
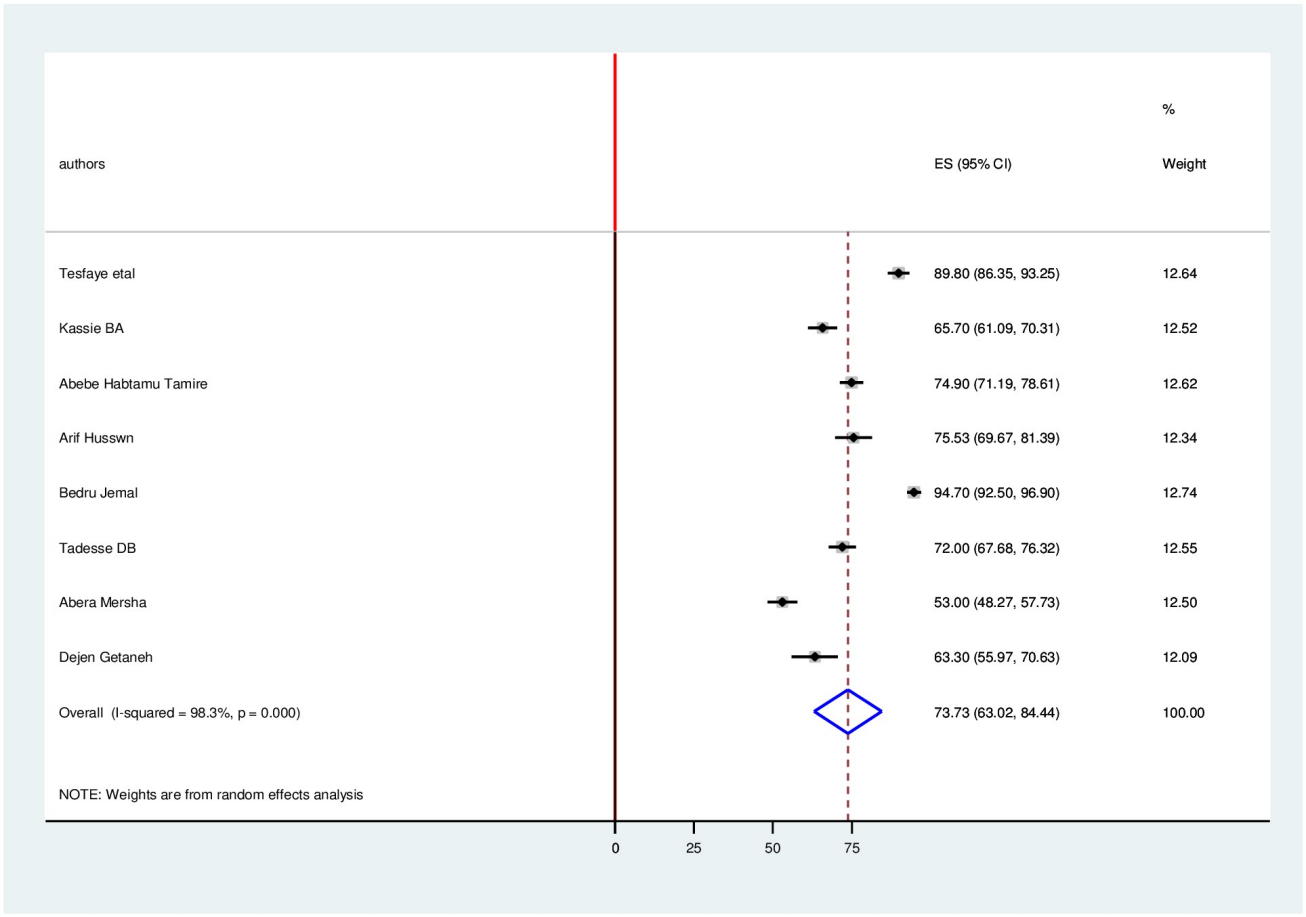
Forest plot for the pooled level of positive attitude towards COVID-19 among health professionals in Ethiopia.

**Fig 7:**
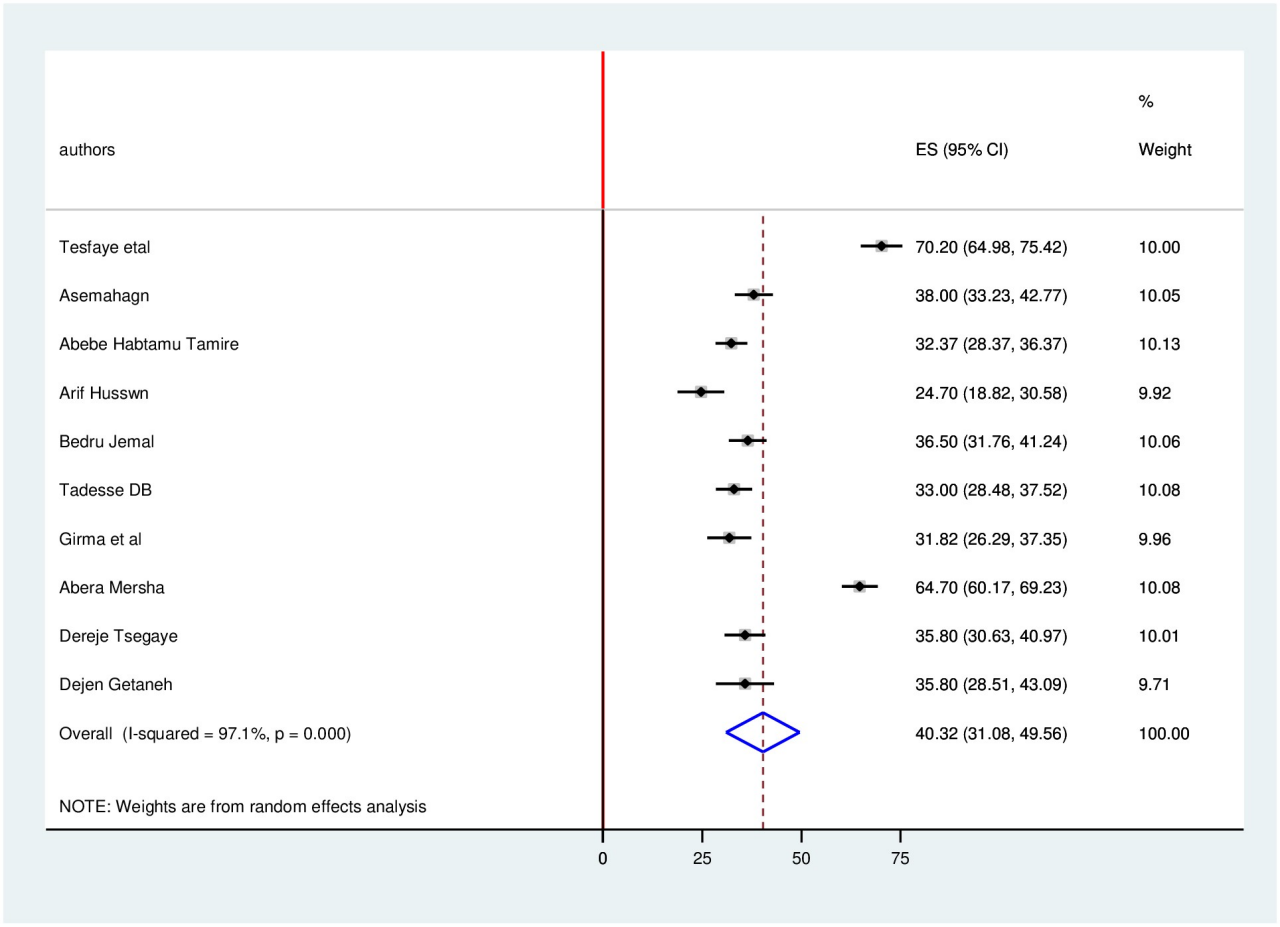
Forest plot for the pooled level of poor practice towards COVID-19 among health professionals in Ethiopia.

The estimated overall level of good knowledge, positive attitude and poor practice in Ethiopia is presented in a forest plot (Figs 5–7). Using the random-effect model, an overall good level of knowledge was found in 79.4% (95% CI: 73.5%-85.2%; I2 = 96%). The pooled estimated level of positive attitude towards COVID-19 was found in 73.73% (95%CI: 63.0%-84.4%; I2 = 98.3%). The estimated level of poor practice towards COVID-19 among health professionals was found in 40.3% (95%CI: 31.1%-49.6%; I2 = 97.1%).

### A leave-out-one sensitivity analysis

A leave-out-one sensitivity analysis was done to evaluate the effect of each study on the pooled level of good knowledge, positive attitude and poor practice by excluding each study step-by-step. The results showed that the excluded study did not bring any significant change to the estimated level of good knowledge, positive attitude and poor practice respectively (Table 2).

**Table 2:** A-leave-out -one sensitivity analysis for knowledge, attitude and practice towards COVID-19 among health professionals in Ethiopia.

***Knowledge related articles***		
**Study omitted**	**Pooled estimate (%)**	**95%CI**
Tesfaye et al	81.94	77.2–87.68
Asemahagn	80.32	74.36–86.28
Kassie BA	79.93	73.74–86.121
Abebe Habtamu Tamire	78.56	71.94–85.18
Arif Husswn	79.33	73.03–85.63
Bedru Jemal	78.45	71.97–84.94
Tadesse DB	79.91	73.71–86.11
Girma et al	78.99	72.58–85.40
Abera Mersha	78.87	72.31–85.44
Dereje Tsegaye	77.95	72.16–83.74
Dejen Getaneh	78.83	72.51–85.14
***Attitude related articles***		
Study omitted	**Pooled estimate (%)**	**95%CI**
Tesfaye et al	71.38	59.02–83.75
Kassie BA	74.87	63.41–86.33
Abebe Habtamu Tamire	73.54	61.12–85.96
Arif Husswn	73.47	61.61–85.31
Bedru Jemal	70.69	61.33–80.07
Tadesse DB	73.96	61.95–85.98
Abera Mersha	76.74	67.07–86.4
Dejen Getaneh	75.16	63.79–86.53
***Practice related articles***		
Study omitted	**Pooled estimate (%)**	**95%CI**
Tesfaye et al	37.01	29.3–44.72
Asemahagn	40.57	30.20–50.94
Abebe Habtamu Tamire	41.2	30.91–51.5
Arif Husswn	42.03	32.38–51.68
Bedru Jemal	40.74	30.39–51.08
Tadesse DB	41.13	30.89–51.37
Girma et al	41.25	31.2–51.3
Abera Mersha	37.58	29.47–45.70
Dereje Tsegaye	40.81	30.58–51.05
Dejen Getaneh	40.8	30.81–50.78

### Subgroup analysis

The subgroup analysis based on the study region and month showed that the level of good knowledge was found in 70.2% in Addis Ababa and 76.1% in Amhara. The level of good knowledge among health professionals was found to be 67.2% and 84.3% in March 2020 and June 2020 respectively. For attitude and practice, 82.4% of study participants in Addis Ababa had a positive attitude towards COVID-19 but 51.2% of HCWS in Addis showed poor practice against COVID-19. Poor practice was higher in June 2020(50.4%) compared to April (35.2%) (Table 3).

**Table 3:** Pooled levels of good knowledge, positive attitude and poor practice towards COVID among health professionals in Ethiopia, 95% CI and heterogeneity estimate with a p-value for subgroup analysis.

Knowledge related articles			
Variable	**Characteristics**	**Pooled level good knowledge 95%(CI)**	**I**^**2**^**(p-value)**
Region	Nationwide	85.903% (80.938, 90.867)	70% (0.68)
	Addis Ababa	70.243% (37.022, 103.464)	99.1% (≤ 0.001)
	Amhara	76.09%(67.99, 84.19)	88.7%(≤ 0.001)
By study month	March	67.151%(55.12, 79.18)	95.0% (≤ 0.001)
	April	83.609%(72.107, 95.11)	97.4% (≤ 0.001)
	May	85.903%(80.938, 90.867)	70%% (0.68)
	June	84.33%(81.407, 87.25)	0.0% (0.0%)
Attitude related articles			
Variable	**Characteristics**	**Pooled level positive attitude 95%(CI)**	**I**^**2**^**(p-value)**
Region	Addis Ababa	82.366% (67.764, 96.967)	97.0%(≤ 0.001)
	Amhara	65.021% (61.12, 68.921)	0.0% (0.587)
Study month	March	75.893% (60.959, 90.827)	97.5% (≤ 0.001)
	June	57.753% (47.689, 67.817)	81.3% (0.021)
Practice related articles			
Variable	**Characteristics**	**Pooled level poor practice 95%(CI)**	**I**^**2**^**(p-value)**
Region	Addis Ababa	51.246% (14.174, 88.319)	99.2% (≤ 0.001)
	Amhara	37.341% (33.35, 41.332)	0.0% (0.621)
	Nationwide	34.385% (29.82, 38.95)	37.1%(0.207)
Study month	March	51.576%(15.121, 88.032)	99.1% (≤ 0.001)
	April	35.157%(31.748, 38.566)	38.9%(0.195)
	May	34.385%(29.82, 38.95)	37.1%(0.201)
	June	50.397%(22.077, 78.717)	97.7%(≤ 0.001)

## Discussion

Ethiopia is one of the most affected countries in East Africa for C0VID-19 and the disease is increasing in the population day by day. When the disease was first recognized in the country, the government declared a five-month state of emergency and established a national COVID-19 response taskforce aimed at mobilizing resources to mitigate transmission of the virus [28]. HCWs were at the forefront of trying to protect themselves and the population from infection [29].

Because of the limited available literature, we conducted this systematic review and meta-analysis to understand better the KAP among HCWs in Ethiopia. We included all available studies conducted between April and June 2020 using a variety of different search engines and were also able to undertake a sub-group analysis assessing the pattern of COVID-related KAP by geographical distribution and by time.

The study showed a generally good knowledge amongst HCWs varying from about 50–90% with a pooled average of nearly 80%. This finding is consistent with a previous study done by Akshaya and his colleagues [30]. The subgroup analysis showed that HCWs from the Amhara region were more knowledgeable compared with those from the Ethiopian capital, Addis Ababa and were more knowledgeable in June compared with March. The latter finding is not surprising and relates to the start of the outbreak in Ethiopia which was in March when there was little information available about the new disease. Over the next few months, the government and other stakeholders released information and guidelines about COVID-19 and front-line experience was also gained. Differences in socioeconomic and educational status and study settings may have also influenced the results.

About three quarters of HCWs also had a positive attitude about the WHO recommendations aimed at mitigating the spread of COVID-19. Positive attitudes were more common in Addis Ababa than in the Amhara region, and this might be due to more exposure to the disease in the capital city. In contrast, positive attitudes decreased between March and June which might have been due to front-line worker experience of difficulties in personal protective measures and the increasing realization that COVID-19 was a serious new infection with considerable morbidity and mortality. The links to faith may also have increased during this time and these may have supplanted the acceptance of WHO recommendations.

The pooled level of poor practice for protective measures against COVID-19 was low at 40%. This was relatively lower than that found in a previous study Bhagavathula and his colleagues [30]. The reasons for this are unclear but may have to do with protective mechanisms conflicting with normal lifestyles, especially amongst the poorer sectors of the population. Ethiopia is a poor country with the majority of its population engaged in hand-to-mouth existence and preventive measures such as home lockdowns are not easily accepted. Furthermore, Ethiopians have strong social ties in which hospitality, eating, and drinking together is a long-standing tradition—this too rides uncomfortably with home lockdowns.

In our study, the variation between studies resulted in a significant between-study heterogeneity. To assess this further, we used a random-effect model as well as a leave one-out-one sensitivity analysis. The results showed that the estimated pooled level of good knowledge, positive attitude and poor practice was robust and not dependent on a single study. We assessed the possible source variability by sub-group analysis using study month and region. The high heterogeneity might be due to differences in the sample population between studies and the short study period for this new emerging virus.

### Strength and limitation of the study

The strengths of the study included the comprehensive search strategy through the different datasets to estimate the national level of knowledge, attitude, and practice towards COVID-19, the involvement of more than one assessor in the quality evaluation and the use of the appraisal process using JBI-MAStARI. The study also had some limitations. These included the fact that measurements for the level of knowledge, attitude and practice were taken from each primary study and operational definitions may have differed between the studies. The absence of a similar previous study makes it difficult to compare our findings with other findings. Finally, our search strategy found limited studies especially no studies from Dire Dawa, Afar, Gambella, Sidama and Benishangul-Gumuz regions and this calls into question the national representativeness of our study.

## Conclusion

Our systematic review and meta-analysis showed that there was a significant gap in knowledge, attitude and practice concerning COVID-19 amongst HCWs in Ethiopia. This is important information and requires that the country step up the provision of accurate and up-to-date information on COVID-19 through the relevant institutions and institute better training and education. Further research is required to determine the KAP of HCWs in other regions of the country and more information about why these gaps in KAP still exist.

## Supporting information

S1 Checklist PRISMA 2009 checklist. (DOC)

S1 File (DOCX)

## Abbreviations

CI: Confidence Interval
COVID-19: Corona Virus Disease
DARE: Database of Abstracts of Review of Effects
Dr: Doctor
EMBASE: Excerpta Medica database
FMOH: Federal Ministry of Health
JBI: The Joanna Briggs Institute
KAP: Knowledge Attitude and Practice
PRISMA: Preferred Reporting Items for Systematic Review and Meta-Analysis
SNNP: South Nations Nationalities and Peoples Region
WHO: World Health Organization
