# Supplementary material for: Knowledge, attitude and practice towards COVID-19 among health professionals in Ethiopia: A systematic review and meta-analysis
Source: PLoS One. 2021 Feb 19;16(2):e0247204. doi: 10.1371/journal.pone.0247204 (PMC7894858; doi:10.1371/journal.pone.0247204)
Supplement: S1 File — (DOCX) [file pone.0247204.s002.docx]

| ***Authors name*** | **Criteria for inclusion** | **Study subject and setting** | **Measure of exposure** | **Identify of confounding factors** | **Strategy to deal with confounding** | **Measure of outcome** | **Objective for measurement of the condition** | **statistical analysis used** | Total | **Covert to percent (100%)** | **Level of quality** |
| --- | --- | --- | --- | --- | --- | --- | --- | --- | --- | --- | --- |
| Tesfaye et al | Yes | Yes | Yes | No | No | Yes | Yes | Yes | 6 | 75 | High |
| Asemahagn | Nc | Yes | Yes | No | Yes | Yes | Yes | Yes | 6 | 75 | High |
| Kassie BA | No | Yes | Yes | No | No | Yes | Yes | Yes | 5 | 62.5 | High |
| Abebe Habtamu Tamire | Yes | Yes | Yes | No | No | Yes | Yes | Yes | 6 | 75 | High |
| Arif Husswn | No | Yes | Yes | No | No | Yes | Yes | Yes | 5 | 62.5 | High |
| Bedru Jemal | No | Yes | Yes | No | Yes | Yes | Yes | Yes | 6 | 75 | High |
| Tadesse DB | Yes | Yes | Yes | No | No | Yes | Yes | Yes | 6 | 75 | High |
| Girma et al | Yes | Yes | Yes | Yes | No | Yes | Yes | Yes | 7 | 87.5 | High |
| Dereje Tsegaye | No | Yes | Yes | No | Yes | Yes | Yes | Yes | 6 | 75 | High |
| Dejen Getaneh | Yes | Yes | Yes | No | Yes | Yes | Yes | Yes | 7 | 87.5 | High |
| Abera Mersha | Yes | Yes | Yes | No | Yes | Yes | Yes | Yes | 7 | 87.5 | High |

Yes=1; no&nc=0 ; Nc=not clear
